# Biomimetic Human Tissue Model for Long-Term Study of *Neisseria gonorrhoeae* Infection

**DOI:** 10.3389/fmicb.2019.01740

**Published:** 2019-07-31

**Authors:** Motaharehsadat Heydarian, Tao Yang, Matthias Schweinlin, Maria Steinke, Heike Walles, Thomas Rudel, Vera Kozjak-Pavlovic

**Affiliations:** ^1^Biocenter, Chair of Microbiology, University of Würzburg, Würzburg, Germany; ^2^Chair of Tissue Engineering and Regenerative Medicine, University Hospital Würzburg, Würzburg, Germany; ^3^Research Center “Dynamic Systems: Systems Engineering” (CDS), Otto von-Guericke-University, Magdeburg, Germany

**Keywords:** 3D tissue model, *Neisseria gonorrhoeae*, small intestinal submucosa scaffold, co-culture, infection

## Abstract

Gonorrhea is the second most common sexually transmitted infection in the world and is caused by Gram-negative diplococcus *Neisseria gonorrhoeae*. Since *N. gonorrhoeae* is a human-specific pathogen, animal infection models are only of limited use. Therefore, a suitable *in vitro* cell culture model for studying the complete infection including adhesion, transmigration and transport to deeper tissue layers is required. In the present study, we generated three independent 3D tissue models based on porcine small intestinal submucosa (SIS) scaffold by co-culturing human dermal fibroblasts with human colorectal carcinoma, endometrial epithelial, and male uroepithelial cells. Functional analyses such as transepithelial electrical resistance (TEER) and FITC-dextran assay indicated the high barrier integrity of the created monolayer. The histological, immunohistochemical, and ultra-structural analyses showed that the 3D SIS scaffold-based models closely mimic the main characteristics of the site of gonococcal infection in human host including the epithelial monolayer, the underlying connective tissue, mucus production, tight junction, and microvilli formation. We infected the established 3D tissue models with different *N. gonorrhoeae* strains and derivatives presenting various phenotypes regarding adhesion and invasion. The results indicated that the disruption of tight junctions and increase in interleukin production in response to the infection is strain and cell type-dependent. In addition, the models supported bacterial survival and proved to be better suitable for studying infection over the course of several days in comparison to commonly used Transwell® models. This was primarily due to increased resilience of the SIS scaffold models to infection in terms of changes in permeability, cell destruction and bacterial transmigration. In summary, the SIS scaffold-based 3D tissue models of human mucosal tissues represent promising tools for investigating *N. gonorrhoeae* infections under close-to-natural conditions.

## Introduction

*Neisseria gonorrhoeae* is a Gram-negative diplococcus and a causative agent of the second most prevalent sexually transmitted infection in the world. More than 78 million new infections per year and the rapid increase in antibiotic resistance make it a serious threat to the public health worldwide (Ohnishi et al., 2011; Wi et al., 2017).

Infection with *N. gonorrhoeae* takes place at the mucosal surfaces of the female cervix and the male urethra, as well as at the anorectal, pharyngeal, and conjunctival mucosa. The infection can ascend, causing salpingitis, pelvic inflammatory disease, and bacteremia. In a small number of cases, the bacteria can cross the endothelial barrier leading to disseminated gonococcal infection (DGI) (Eisenstein and Masi, 1981).

*N. gonorrhoeae* contain different types of virulence factors including lipooligosaccharide (LOS), type IV pili, opacity-associated (Opa) proteins, and an outer membrane porin PorB, which enable the bacteria to attach to the epithelial cells, invade them, or survive in the presence of serum. LOS are modified through sialylation during infection and play a role in molecular mimicry, because they resemble host glycosphingolipids (Mandrell and Apicella, 1993; Moran et al., 1996). Pili mediate adhesion to epithelial cells, twitching motility, and microcolony formation of *N. gonorrhoeae* (Punsalang and Sawyer, 1973; Craig et al., 2004). After the initial contact, Opa proteins mediate an efficient invasion into the host cells (Makino et al., 1991). Additionally, *N. gonorrhoeae* express one of the two subtypes of PorB, PorB_IA_, or PorB_IB_, of which PorB_IA_ has been implicated in gonococcal resistance to serum and in DGI (Ram et al., 1999; Rechner et al., 2007).

The immune reaction upon infection differs between different tissues, as well as between females and males (Edwards and Apicella, 2004). In the male urethra primary cell model, the levels of interleukin (IL)-6 and -8 increase upon challenge with gonococci (Harvey et al., 2002). In female cervical cells, upregulation of IL-8 and IL-6, as well as of the intercellular adhesion molecule 1 (CD54), and the non-specific cross-reacting antigen (CD66c) has likewise been observed (Fichorova et al., 2001). There is evidence for upregulation of IL-8, tumor necrosis factor alpha (TNFα) and chemokine (C-C motif) ligand 20 (CCL20), but not of IL-6 in endometrial cell models, which requires living bacteria and appears to be pilus-dependent (Christodoulides et al., 2000; Łaniewski et al., 2017). Whereas TNFα secretion was increased upon infection independently of pilus expression, pilus-positive gonococci caused an increase in IL-8 and suppression of IL-6 secretion (Christodoulides et al., 2000).

*N. gonorrhoeae* has also been shown to cause a disruption of the apical junction of infected cells. Apical junction complexes enable strong adhesion between epithelial cells and serve to protect the integrity of the underlying compartments. They include the apical most tight junction, also known as zonula occludens, and the adherens junction, or zonula adherens (Wang and Margolis, 2007). Upon gonococcal infection, the redistribution of adherens junction proteins E-cadherin and β-catenin takes place. Tight junction proteins Occludin and Zonula Occludens Protein 1 (ZO-1) were reported not to be modified in non-polarized cells (Rodríguez-Tirado et al., 2012), whereas a redistribution of ZO-1 was observed for polarized cells infected with gonococci (Edwards et al., 2013). Since the formation of apical junction is related to cell polarization, it is obvious that the usage of appropriate models when studying gonococcal infection greatly affects the relevance of the obtained data.

Regarding models, human cancer cell lines of epithelial tissues have often been used to investigate the host cell side during the contact with *N. gonorrhoeae*, although animal studies on chimpanzee and especially transgenic mouse opened new opportunities for understanding the gonococcal infection (Rice et al., 2017). These models were successful in recreating the early stages of infection, but show differences in susceptibility and immune response (Packiam et al., 2010).

The examples of human tissue and polarized cell models include endocervical tissue explants and epithelial cells grown on Transwell® inserts, which were used to show effects of *N. gonorrhoeae* on immune response, disruption of apical junction and shedding of epithelial cells (Buckner et al., 2011; Stein et al., 2015; Wang et al., 2017). Furthermore, three-dimensional (3D) models of human endometrial epithelial tissue in rotating wall vessel bioreactor could be successfully infected for studying gonococcal pathogenicity (Łaniewski et al., 2017). Apart from tissue explants, all other models are limited in terms of morphology or absence of other cell types besides epithelial cells.

To generate 3D tissue models, natural scaffolds such as decellularized tissue have been successfully used. Such scaffolds keep extracellular matrix characteristics of the native organ and provide suitable conditions for cell proliferation and differentiation (Costa et al., 2017). Among them, porcine small intestinal submucosa (SIS) scaffold has been widely used in many studies in order to establish a human tissue barrier for drug delivery investigations (Liu et al., 2017; Schweinlin et al., 2017). This type of biological scaffold supported by cell crowns is comparable to the commonly used Transwell® insert system, with two separated compartments being present (Schweinlin et al., 2016).

In this study, we have developed novel tissue models based on SIS scaffold that are comprised of two types of cells, primary human dermal fibroblasts (HDFib) and target epithelial cells of *N. gonorrhoeae*. We show that SIS-based models demonstrate improved charateristics in comparison to the Transwell® models in terms of tissue permeability and resistance to bacterial infection. With the aid of SIS models, we show that the effects of *N. gonorrhoeae* on the tissue, which we measure in terms of bacterial transmigration, tissue permeabiliy and cytokine response, depend both on the type of tissue, as well as on the pathogenicity determinants present in the bacterial strain. Taken together, our models represent a solid and reproducible tool for studying the interaction of bacteria with host tissue during long-term gonococcal infection.

## Results

### Establishment and Characterization of SIS-Based 3D Tissue Models

**Figure 1:**
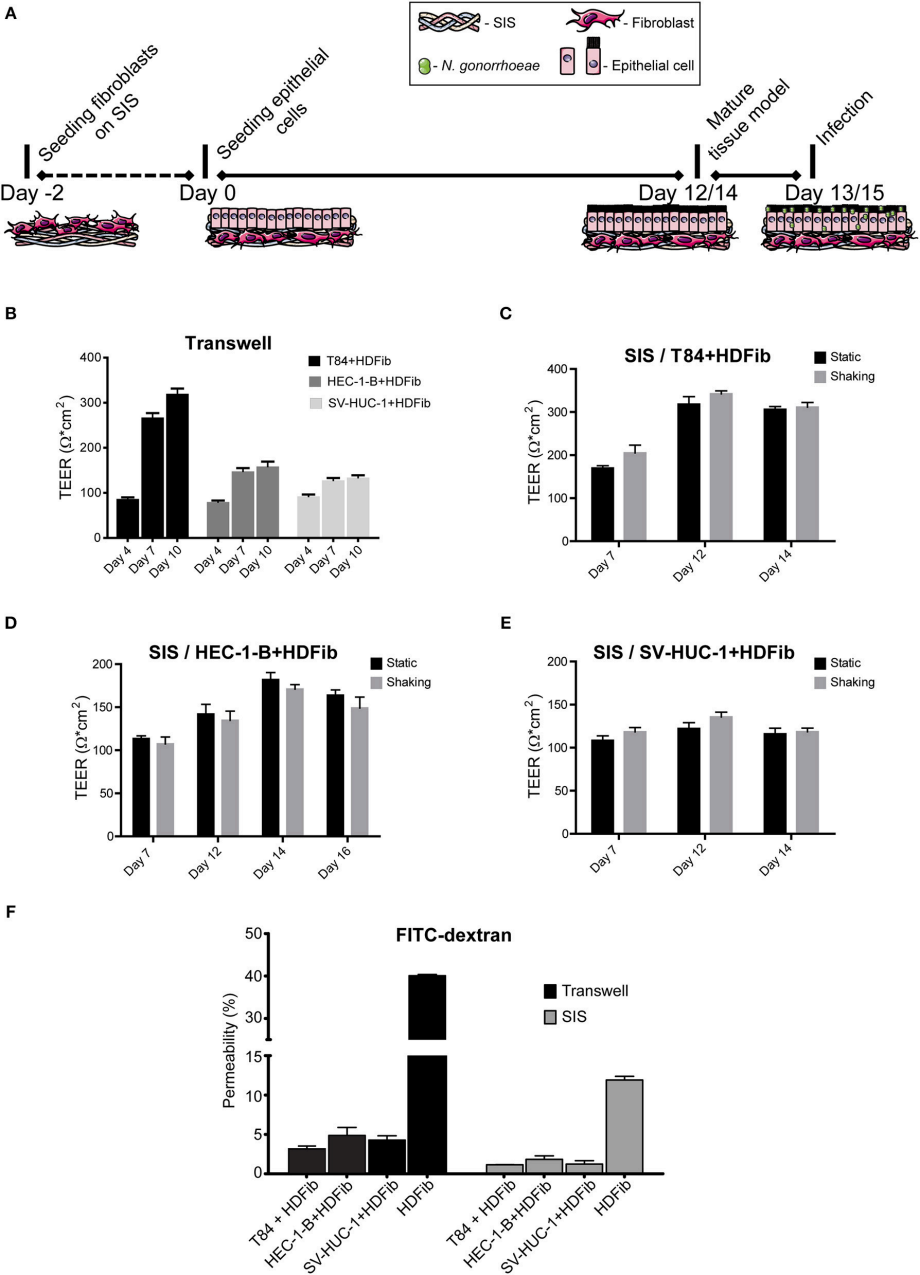
Establishment of mucosal tissue models on porcine small intestinal submucosa (SIS) scaffold. **(A)** Schematic representation of the preparation of epithelial/fibroblast co-culture tissue models on SIS using cell crowns with an optional infection step. **(B)** Transwell® inserts were coated with collagen on both sides of the membrane. HDFib were seeded on the basal side, followed by the seeding of epithelial cells on the apical side 48 h later (day 0). TEER was measured at indicated time points. **(C–E)** HDFib and specified epithelial cells were seeded on the SIS as described in **(A)**. Tissue models were grown either under static conditions, or shaking using an orbital shaker. TEER was measured at indicated time intervals. **(F)** Tissue models on Transwell® or SIS were generated as described in **(A,B)**. Permeability was measured by 4 kDa FITC-dextran assay at day 10 for Transwell® models, at day 12 for SIS models for T84 and SV-HUC-1 cells and at day 14 for HEC-1-B cells. The graph shows fluorescence intensity from the lower compartment normalized against the fluorescence intensity obtained when empty Transwell® or SIS were used. All graphs represent mean values ± SD from at least three independent replicates.

We established different mucosal tissue models for gonococcal infection: a tight epithelial barrier model with highly polarized cells based on the colon carcinoma T84 cell line, a model representing female reproductive tract mucosal tissue using endometrial adenocarcinoma cell line HEC-1-B, and a model for the male urogenital tissue consisting of the immortalized uroepithelial cell line SV-HUC-1. Epithelial cells were seeded on SIS scaffold that was previously populated with HDFib for 2 days to obtain a biomimetic model (Figure 1A, Supplemental Figure 1). Tissue models were allowed to mature over the course of 12 to 14 days, and maturation was followed by measuring parameters such as transepithelial electrical resistance (TEER) or barrier permeability using FITC-dextran assay (Figures 1C–F). In comparison, we seeded the same epithelial cells on the apical side in combination with HDFib cells seeded on the basal side of the Transwell® inserts (Figure 1B). The TEER values for the Transwell® models showed an increase with time, reaching the maximum of ~320 Ω^*^cm^2^ for T84, ~160 Ω^*^cm^2^ for HEC-1-B and ~130 Ω^*^cm^2^ for SV-HUC-1 on day 10 of cultivation (Figure 1B). The SIS tissue models showed comparable values of TEER, however only after 12 days for T84 and SV-HUC-1 cells and 14 days for HEC-1-B cells. The introduction of shear stress to models through cultivation on the orbital shaker only slightly improved the barrier formation and cell polarization, and in the case of HEC-1-B cells had even a slightly negative effect (Figures 1C–E). For this reason, all subsequent SIS models were grown in static culture. The permeability of both Transwell® and SIS models was tested using the FITC-dextran permeability assay. Mature SIS models with epithelial cells were much less permeable with 1–2% permeability in comparison to only SIS scaffold with HDFib (12% permeability), but also when compared to the respective Transwell® models (3–5% permeability) (Figure 1F).

**Figure 2:**
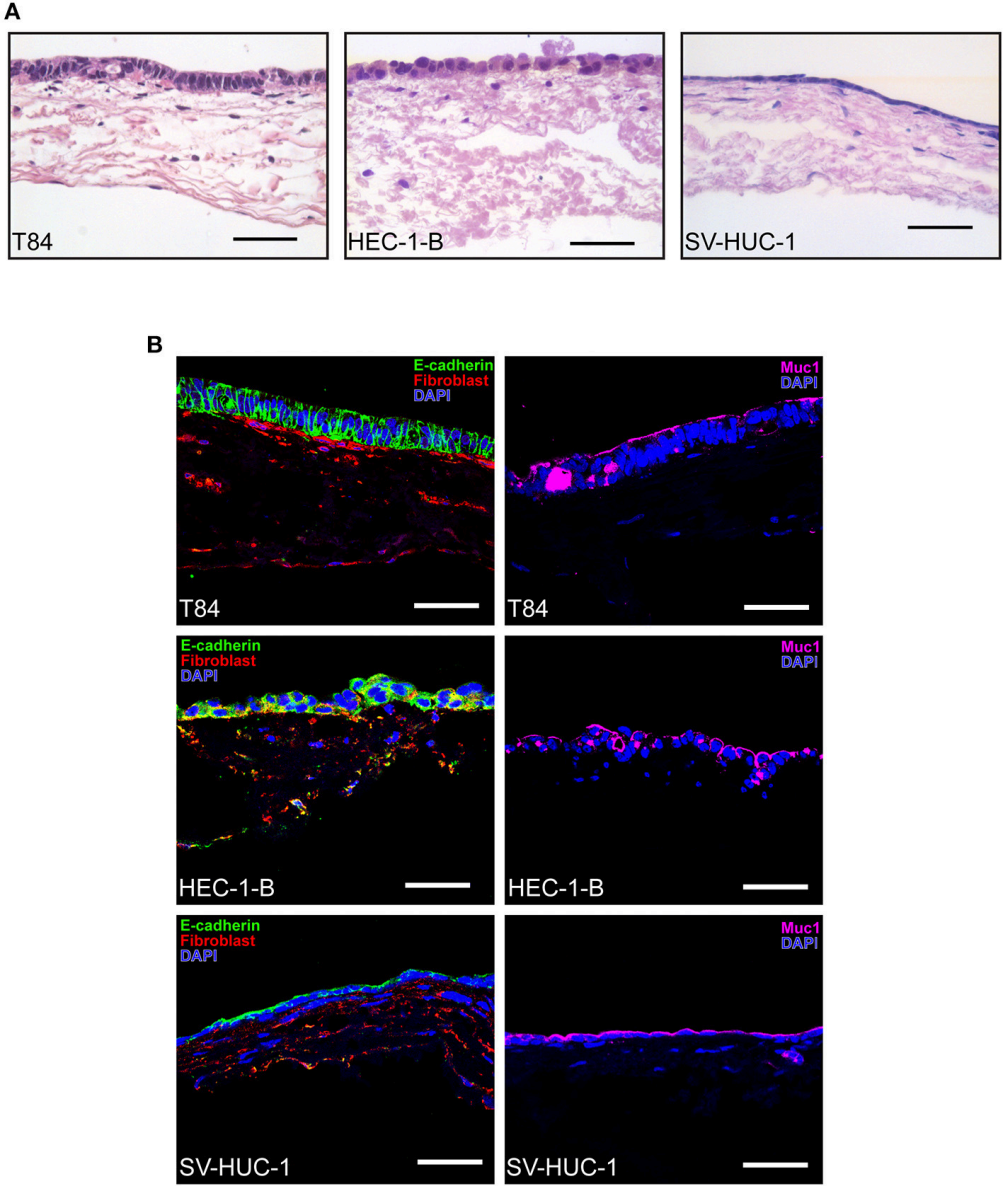
Histological characterization of SIS mucosal tissue models. **(A)** Epithelial/fibroblast co-culture tissue models were grown as described in Figure 1A for 12 days (T84 and SV-HUC-1 cells) or 14 days (HEC-1-B cells). Tissue models were fixed, paraffin embedded, sectioned, and hematoxylin and eosin staining was performed. **(B)** Tissue models were prepared as in **(A)** and decorated with anti-E-cadherin, anti-mucin 1 (Muc1) and anti-fibroblast antibodies. Cell nuclei were stained with DAPI. Scale bar is 50 μm.

Histological characterization of SIS tissue models using hematoxylin eosin (HE) staining and immunofluorescence on tissue sections made after paraffin embeding showed a monolayer of epithelial cells located on the appical side of the scaffold, where occasionally fibroblasts could be observed growing within the SIS scaffold (Figure 2). T84 polarized to a tall columnar phenotype and were stained with E-cadherin and mucin 1 (Muc1) antibodies, demonstrating cell-cell contacts and mucus production. Mucus could be occasionally identified in large vesicles inside cells. HEC-1-B cells on the other hand did not always grow in a monolayer and were not as polarized as T84 cells, consistent with what has been described previously (Edwards et al., 2013). In addition to Muc1, they were also positive for E-cadherin and anti-fibroblast staining, which is characteristic for endometrial adenocarcinoma cells that demonstrate epithelial-to-mesenchymal transition (Mirantes et al., 2013). Finally, SV-HUC-1 cells appeared as a flat monolayer positive for both E-cadherin and Muc1 staining (Figure 2).

**Figure 3:**
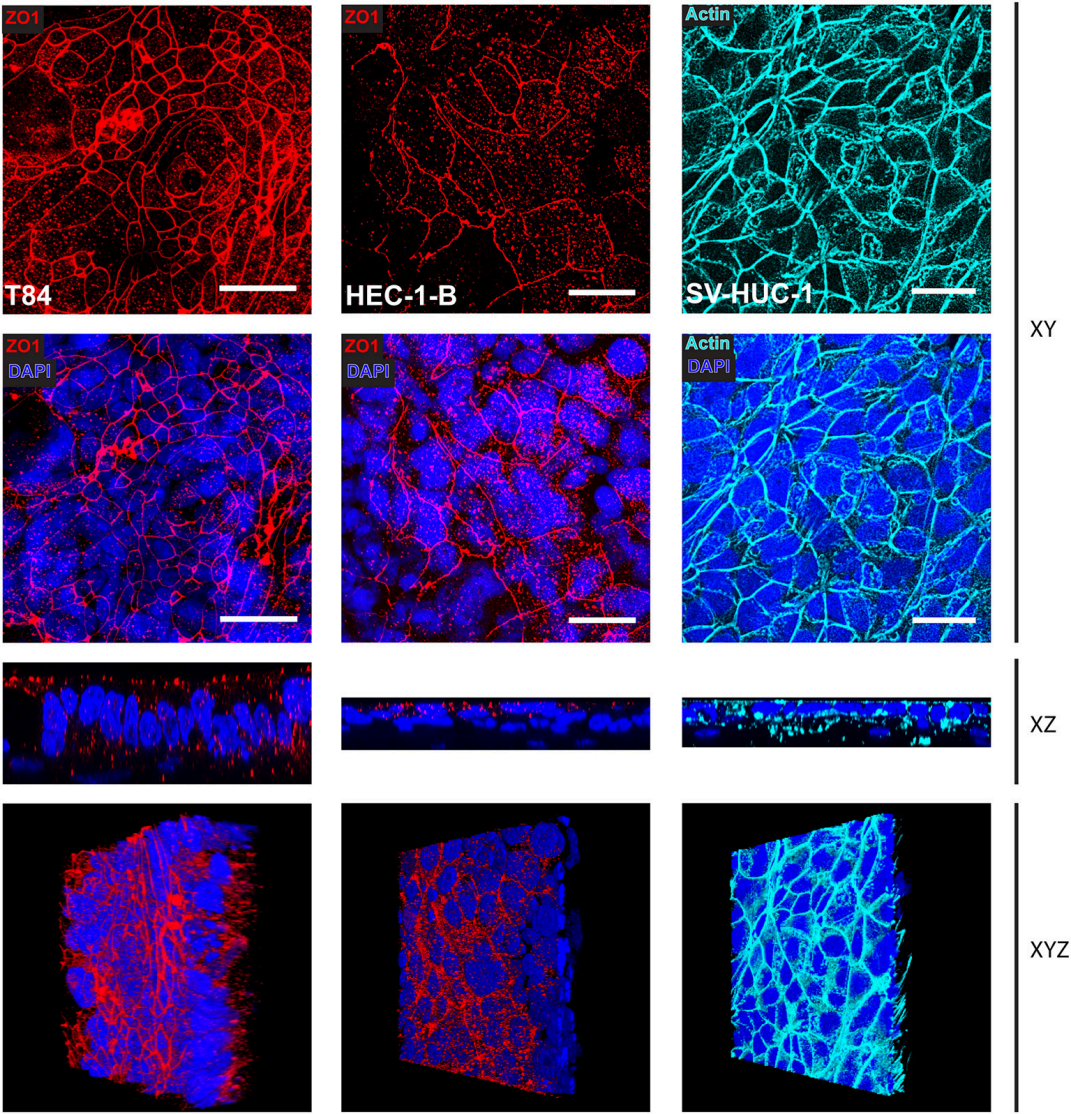
Confocal microscopy of SIS mucosal tissue models. Epithelial/fibroblast co-culture tissue models were cultured for 12 days (T84 and SV-HUC-1 cells) or 14 days (HEC-1-B cells) as represented in Figure 1A. Tissue models were fixed on cell crowns, then decorated using anti-zonula occludens 1 (ZO1) antibody, phalloidin (actin), and DAPI. Z-stacks were made using fluorescence confocal microscope from the top of the epithelial layer to the beginning of collagen scaffold and reconstructed using FIJI. Shown are Z-projections (XY), orthogonal view (XZ), or a snapshot of a reconstructed 3D image (XYZ). Scale bar is 25 μm. See also Supplemental Movies 1–3.

We adapted the technique for immunofluorescence staining after paraformaldehyde fixation followed by confocal microscopy to SIS models and analyzed the morphology of the tissues, as well as the distribution of the tight junction marker ZO1. Whereas, T84 cells showed a strong staining for ZO1 present at the very apex of the cell layer (Figure 3, left hand panels), HEC-1-B cells were less clearly stained, although still positive for ZO1 (Figure 3, middle panels). SV-HUC-1 cells presented a continuous layer as visualized by phalloidin staining of actin (Figure 1, right hand panels). The ZO1 staining was also present, but was hard to visualize by confocal microscopy on the flat sheet that SV-HUC-1 cells formed (Supplemental Figure 2). The 3D reconstruction of stacks of confocal images showed continuous layers of tall (T84), medium tall (HEC-1-B) and flat (SV-HUC-1) epithelial cells (Supplemental Movies 1–3).

**Figure 4:**
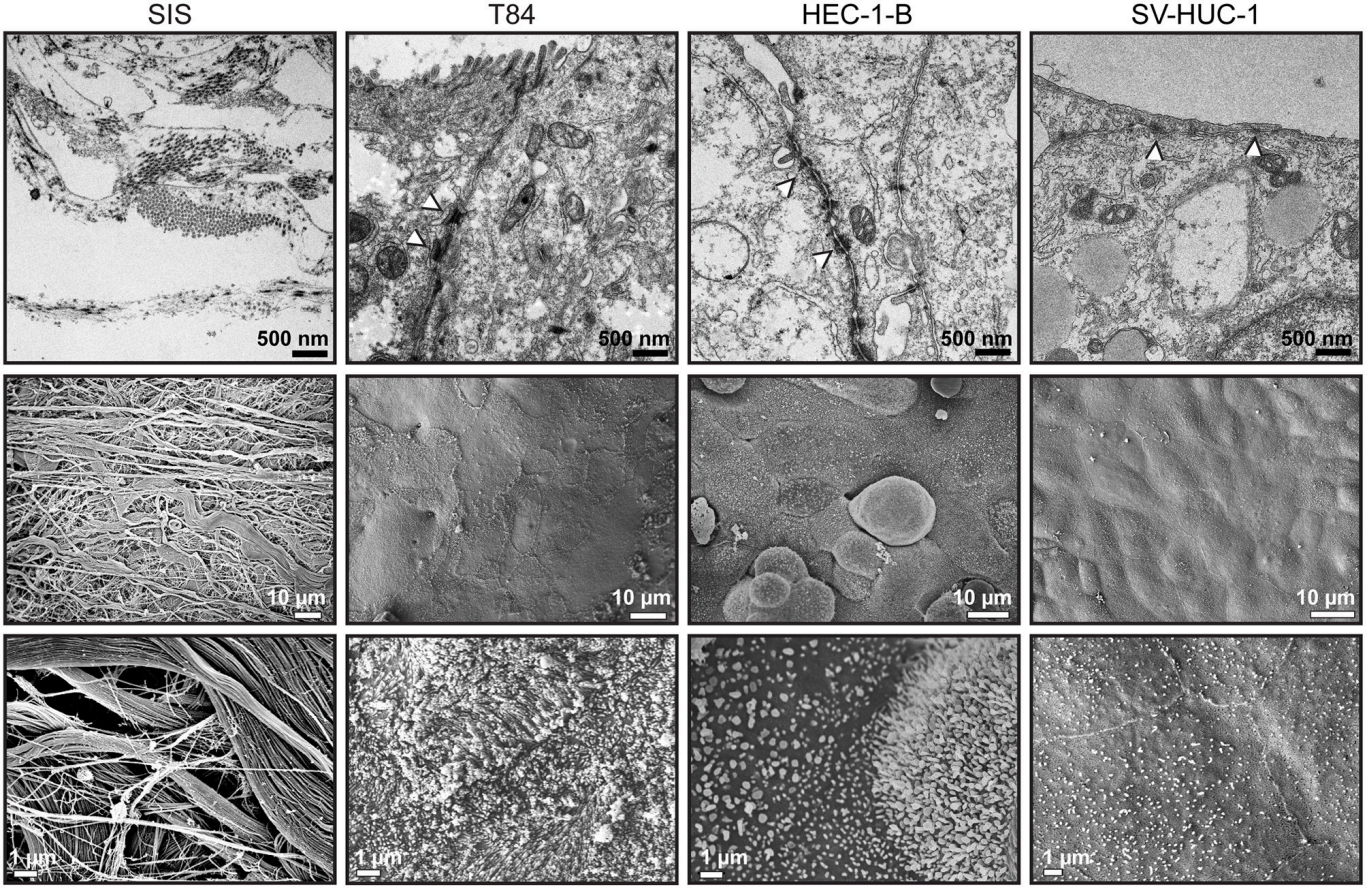
Transmission and scanning electron microscopy of SIS mucosal tissue models. Tissue models on SIS were generated as described in Figures 1A, 2, 3, fixed with glutaraldehyde and either embedded in epoxy resin and analyzed by transmission electron microscopy (upper panels) or analyzed by scanning electron microscopy (middle and lower panels).

We were next interested in the ultrastructure of the SIS tissue models and analyzed them using transmission (TEM) and scanning (SEM) electron microscopy. The SIS scaffold was seen as a mesh of fibers (Figure 4, left hand panels). TEM showed formation of apical junctions between the epithelial cells (Figure 4, white arrowheads). T84 and SV-HUC-1 cells appeared as a continuous monolayer, whereas groups of HEC-1-B cells occasionaly protruded from the underlying monolayer. Under greater magnification, we observed microvilli formation on the apical surface of the cells. The microvilli were most pronounced on T84 cells, followed by HEC-1-B cells, whereas on SV-HUC-1 cells they were fewer and shorter (Figure 4). At the edges of the areas where cells are seeded, a transition to the underlying scaffold can be seen, revealing continuous contacts between the epithelial cells (Supplemental Figure 3A).

### The Course of Gonococcal Infection Depends on the Bacterial Strain and Tissue Type

After establishing SIS tissue models, we next compared the infection with various strains of *N. gonorrhoeae*, also in comparison to standard Transwell® models. For infection, we used different laboratory derivatives, as well as one clinical isolate. The MS11 strain derivatives N927 and N924 were selected to be Opa^−^ and Pili^−^ and differed only in the expressed PorB variant—N924 expresses PorB_IB_, whereas N927 expresses PorB_IA_. For MS11 wildtype and the related ΔOpa derivative that is devoid of any Opa proteins (Stein et al., 2015) Pili^+^ colonies were selected, however subsequent western blot showed that the bacteria used for infection were unpiliated, since they did not express pilin (data not shown). VP1 is a clinical isolate expressing PorB_IA_ (Makino et al., 1991) and Opa^−^ and Pili^−^ colonies were selected for this strain. Measurements show comparable growth of all strains and derivatives, with VP1 and N927 growing slighly slower than the rest (Supplemental Figure 4A). The infection was performed in HEPES medium, which is phosphate and serum free and permits host cell invasion also via the phosphate sensitive interaction with PorB_IA_. As a readout for infection, we assessed the number of bacteria that transmigrated through the tissue model and could be detected in the medium on the basal side. Likewise, we measured the changes in permeability of models to FITC-dextran upon infection.

**Figure 5:**
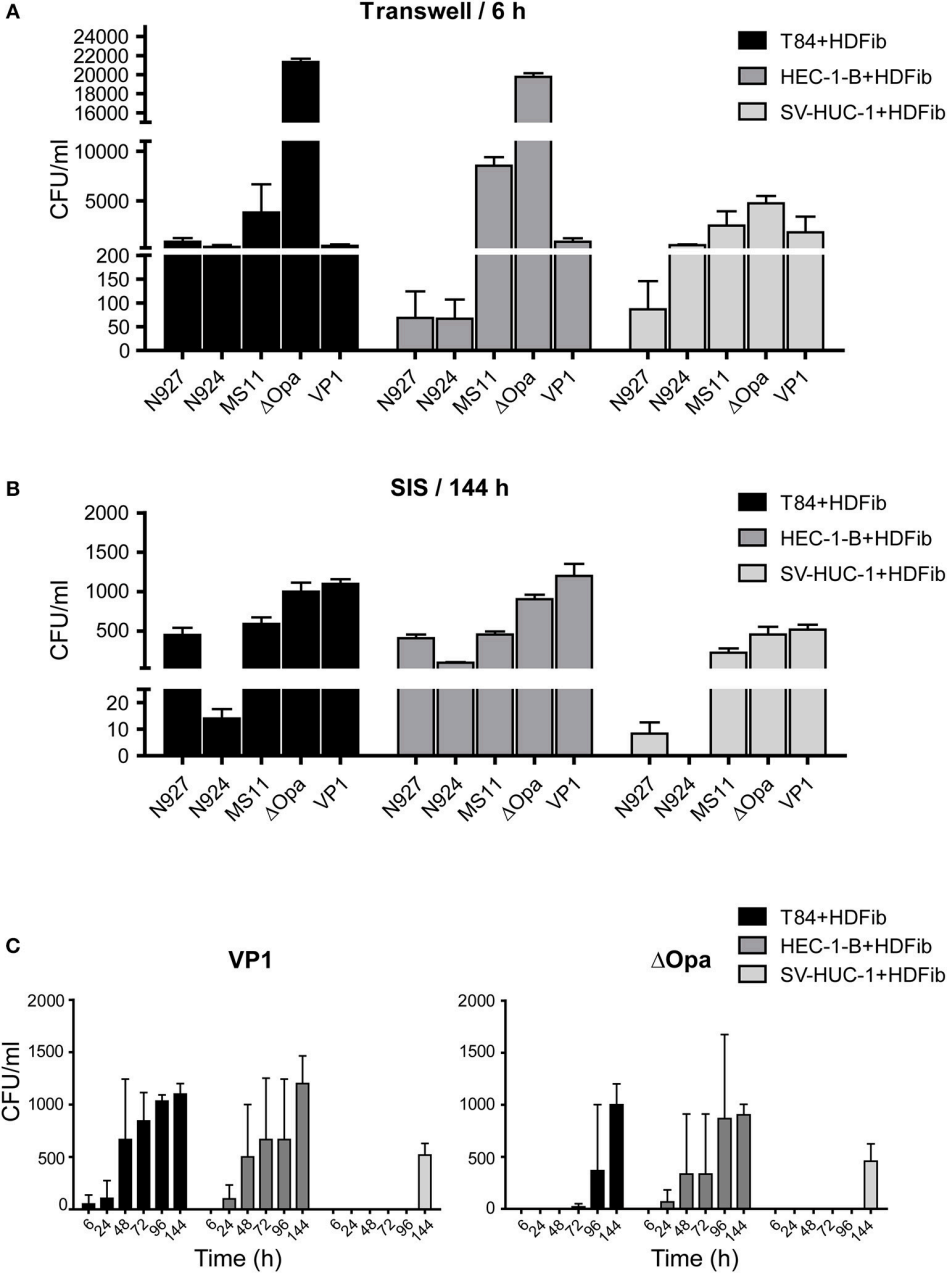
Bacterial transmigration through the Transwell® and SIS tissue models. **(A)** Tissue models on Transwell® inserts were prepared using HDFib and designated epithelial cell lines as described for Figure 1. Eleven days after seeding of the epithelial cells, models were infected with indicated strains of *N. gonorrhoeae* at MOI 20 for 24 h and the number of bacteria in the lower compartment was determined by plating and counting colony forming units (CFU). **(B)** SIS tissue models were generated as described in Figure 1A. Thirteen (T84 and SV-HUC-1 cells) or 15 (HEC-1-B cells) days after seeding of epithelial cells, models were infected with indicated strains of *N. gonorrhoeae* at MOI 20 for 144 h (6 days) and the number of bacteria present in the medium from the lower compartment was determined. **(C)** SIS tissue models were prepared as in **(B)**, infected with *N. gonorrhea* VP1 or ΔOpa at MOI 20 and the number of bacteria in the medium from the lower compartment was determined at indicated times post infection. All graphs represent mean values ± SD from at least three independent replicates.

When we infected Transwell® models, we were able to collect a large number of bacteria on the basal side already 6 h after infection. The largest number of transmigrated bacteria was obtained for ΔOpa bacteria for all three types of epithelial cells, similar to what has been previously reported (Stein et al., 2015). Interestingly, SV-HUC-1 cells were the least permeable for ΔOpa bacteria in comparison to the other two types of epithelial cells (Figure 5A). In case of the SIS tissue models, for all bacteria except VP1 and ΔOpa we could detect a relatively small number of transmigrating bacteria only after 6 days (144 h) of infection. The least efficient in transmigration were N924, and although ΔOpa bacteria still transmigrated in greater numbers than the corresponding wild type, VP1 was more efficient than ΔOpa (Figure 5B). Depending on the tissue, larger numbers of transmigrating bacteria for VP1 and ΔOpa could be observed already after 48 h of infection. SV-HUC-1 cells were in this case also presenting the most resistant barrier for bacterial transmigration (Figure 5C). When analyzed by SEM, we could confirm successful adhesion of bacteria to the cell surface and the elongation of microvilli (Supplemental Figure 3B). Interestingly, western blot analysis of the gonococcal Opa phenotype before and after infection of the SIS scaffold models showed that even though the selection for Opa^−^ phenotype was successful for all strains and derivatives except VP1, transmigrated gonococci largely switched to the Opa^+^ phenotype, with the exception of ΔOpa and VP1. We also observed that the exposure of bacteria to the SV-HUC-1 models led to the lower expression of Opa proteins and in the case of VP1 surprisingly led to the loss of Opa expression altogether (Supplemental Figure 4B). Importantly, the differences and delayed transmigration of bacteria through the SIS models was not due to the association of bacteria with the scaffold, because the empty SIS scaffold, as well as the SIS scaffold populated with fibroblasts for 2 days, were not presenting much of a barrier. We could collect high numbers of bacteria from the basolateral compartment already 30 min after infection and in amounts generally similar for all strains and derivatives (Supplemental Figure 5). In conclusion, SIS models seem to present a better barrier to bacterial transmigration in comparison to Transwell® models. In addition, the efficiency of transmigration of different gonococcal strains and derivatives differs between Transwell® and SIS models.

**Figure 6:**
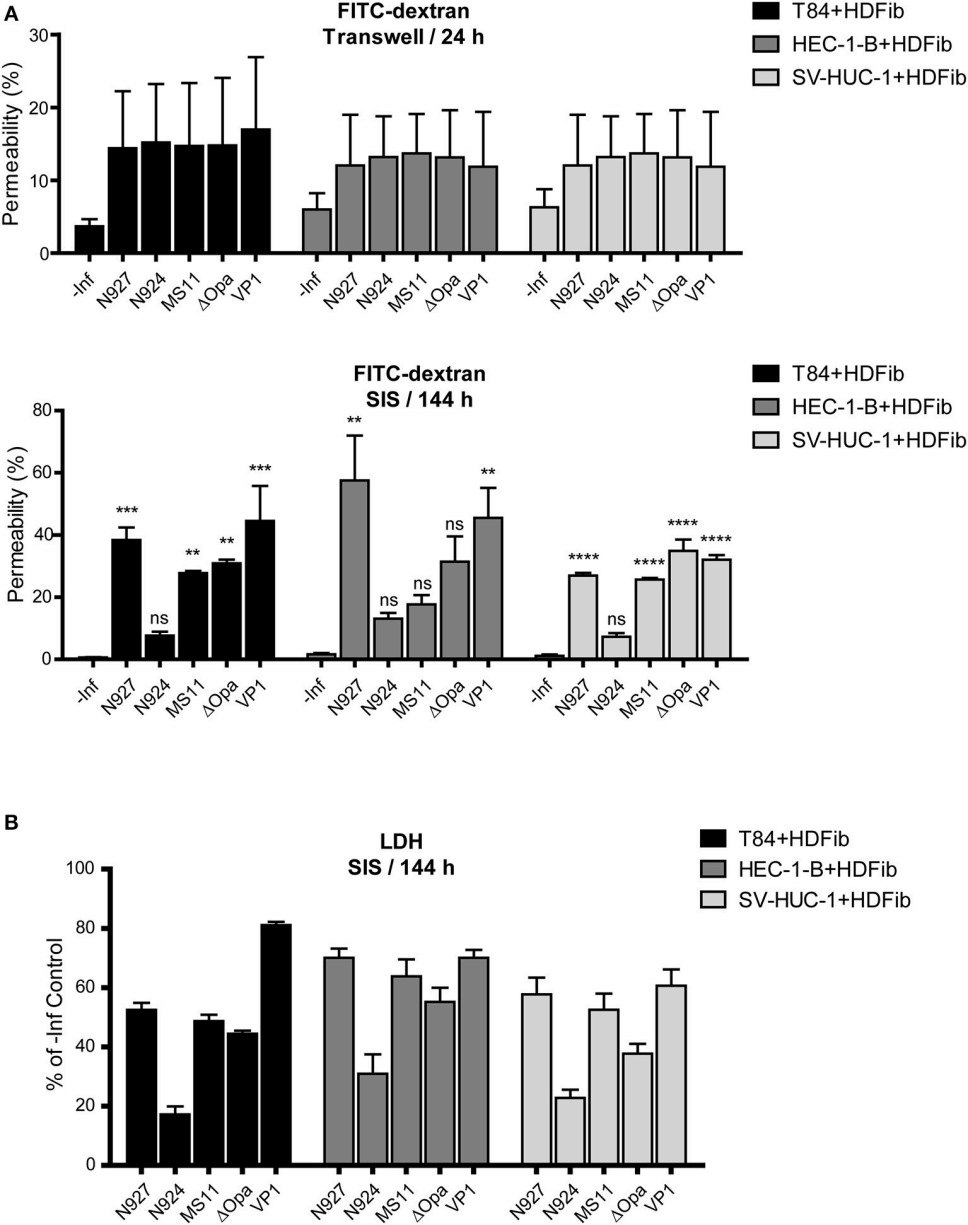
Barrier permeability and cell death in mucosal tissue models upon infection with *N. gonorrhoeae*. **(A)** Tissue models were prepared and infected with different strains of *N. gonorrhoeae* for 24 h (Transwell® models) or 144 h (SIS models) as described for the Figure 5. Permeability of the models was measured by 4 kDa FITC-dextran assay. The graphs represent mean values ± SD from at least three independent replicates. Statistical significance of permeability change in comparison to non-infected control was tested by one-way ANOVA: ^*ns*^*p* > 0.05, **p ≤ 0.01, ***p ≤ 0.001, ****p ≤ 0.0001. **(B)** SIS scaffold models infected for 144 h were assayed for cytotoxicity using lactate dehydrogenase (LDH) assay. The graphs represent mean values ± SD from at least three independent replicates.

Permeability measurements of Transwell® models showed an increase of permeability to ~15% upon infection, which was independent of cell type and of bacterial strain used (Figure 6A, upper panel). For SIS models, longer times were necessary for a significant change in permeability after infection (Supplemental Figure 6). After 6 days (144 h), at the time point when we could detect larger amounts of bacteria traversing the SIS model, the permeability also increased, in some cases to over 50%. Interestingly, we could see clear differences in induction of SIS model permeability for different bacterial strains, which often, but not always, correlated to the number of transmigrated bacteria. VP1 and N927 strains showed the greatest capacity for increasing SIS model permeability (Figure 6A, lower panel). To test if the permeability of SIS models was related to cell lysis, we measured the lactate dehydrogenase (LDH) activity in the supernatant in comparison to the non-infected control. In general, the damage to the cells corellated with increased permeability of models and the number of bacteria that crossed the tissues (Figure 6B). Taken together, our results indicate that gonococci harboring PorB_IA_ cause the greatest damage to the SIS tissue models, which is coupled to the increased permeability and a comparably high number of traversing bacteria in the case of VP1, but not N927.

**Figure 7:**
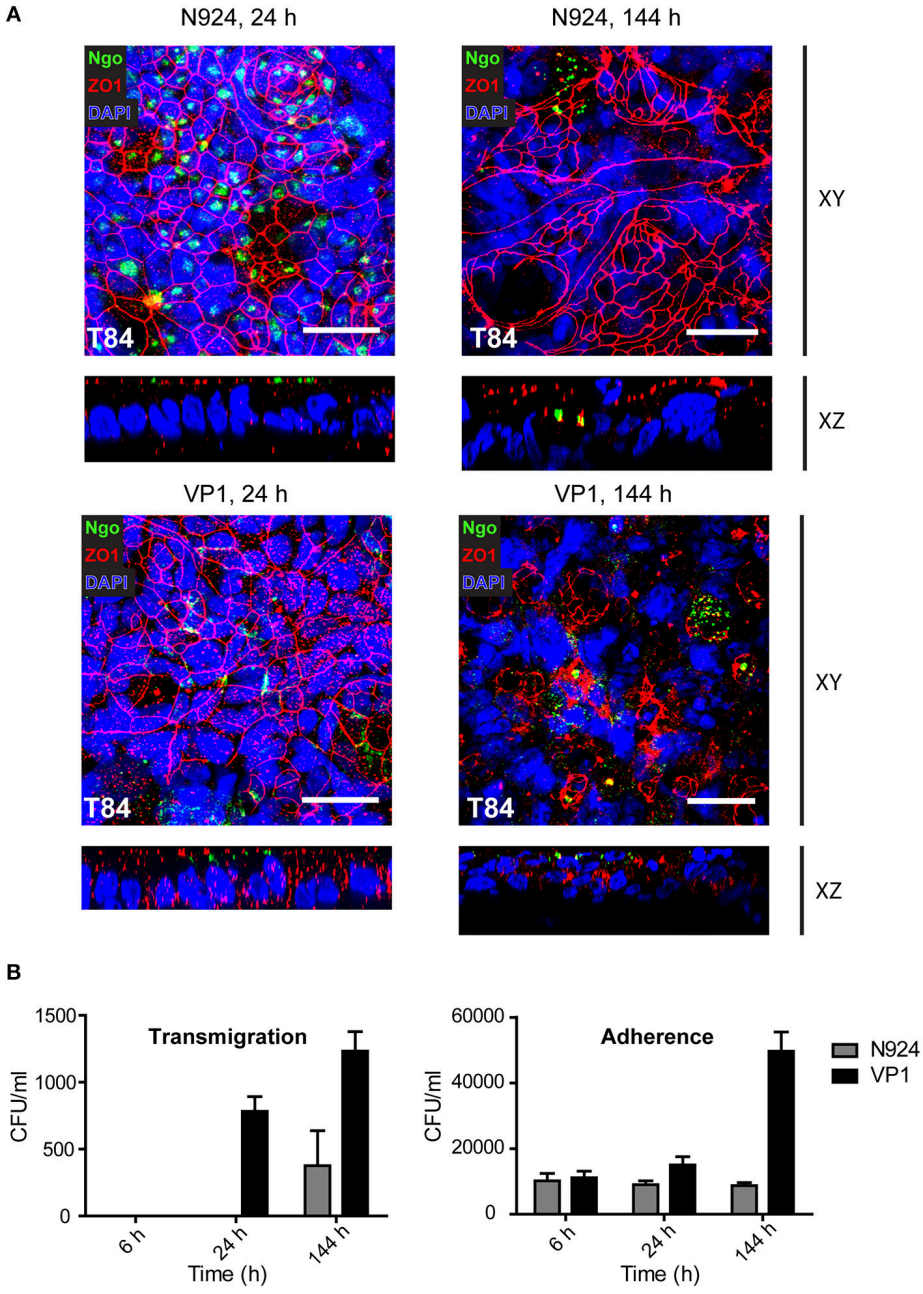
Confocal microscopy and analysis of infected T84 SIS tissue models. **(A)** Tissue models with T84 epithelial cells were prepared and infected with N924 or VP1 strains of *N. gonorrhoeae* for 24 or 144 h, as described for the Figure 5. The infected models were fixed on cell crowns and decorated using anti-zonula occludens 1 (ZO1) and anti-*N. gonorrhoeae* antibody, and DAPI. Z-stack images were made using fluorescence confocal microscope beginning at the top of the epithelial layer to the collagen scaffold. The images were analyzed and reconstructed using FIJI. Shown are Z-projections (XY) and orthogonal view (XZ). Scale bar is 25 μm. See also Supplemental Movies 4–7. **(B)** Tissue models with T84 epithelial cells as in **(A)** were infected with N924 or VP1 strains of *N. gonorrhoeae* for 6, 24, or 144 h. Number of transmigrated bacteria in the basolateral medium, and adherent bacteria upon saponin solubilization of the tissue were assessed by plating and counting colony forming units (CFU). The graphs represent mean values ± SD from at least three independent replicates.

Using confocal microscopy we analyzed the early and late stages of infection of T84 SIS models with the least and the most aggressive bacteria, N924 and VP1, respectively. After 24 h, the cell layer and tight junctions appeared still conserved, whereas at the later stage of infection after 144 h we observed significant cell lysis and destruction of tight junctions in SIS models infected by VP1, but not so much when N924 gonococci were used. Interestingly, whereas N924 bacteria seem to be distributed more in the middle of the cells, VP1 localize in the areas of cell-cell contacts. In the orthogonal views of the samples, we observe bacteria only at the surface of the models (Figure 7A). We repeated the experiments, this time simultaneously assessing the number of transmigrated, as well as adherent, bacteria. VP1 strain showed again high capacity for transmigration, and the number of VP1 bacteria associated with the model greatly increased after 6 days of infection, whereas the number of N924 bacteria remained approximately the same (Figure 7B).

### Tissue Response to Infection Is Strain- and Cell Type-Dependent

**Figure 8:**
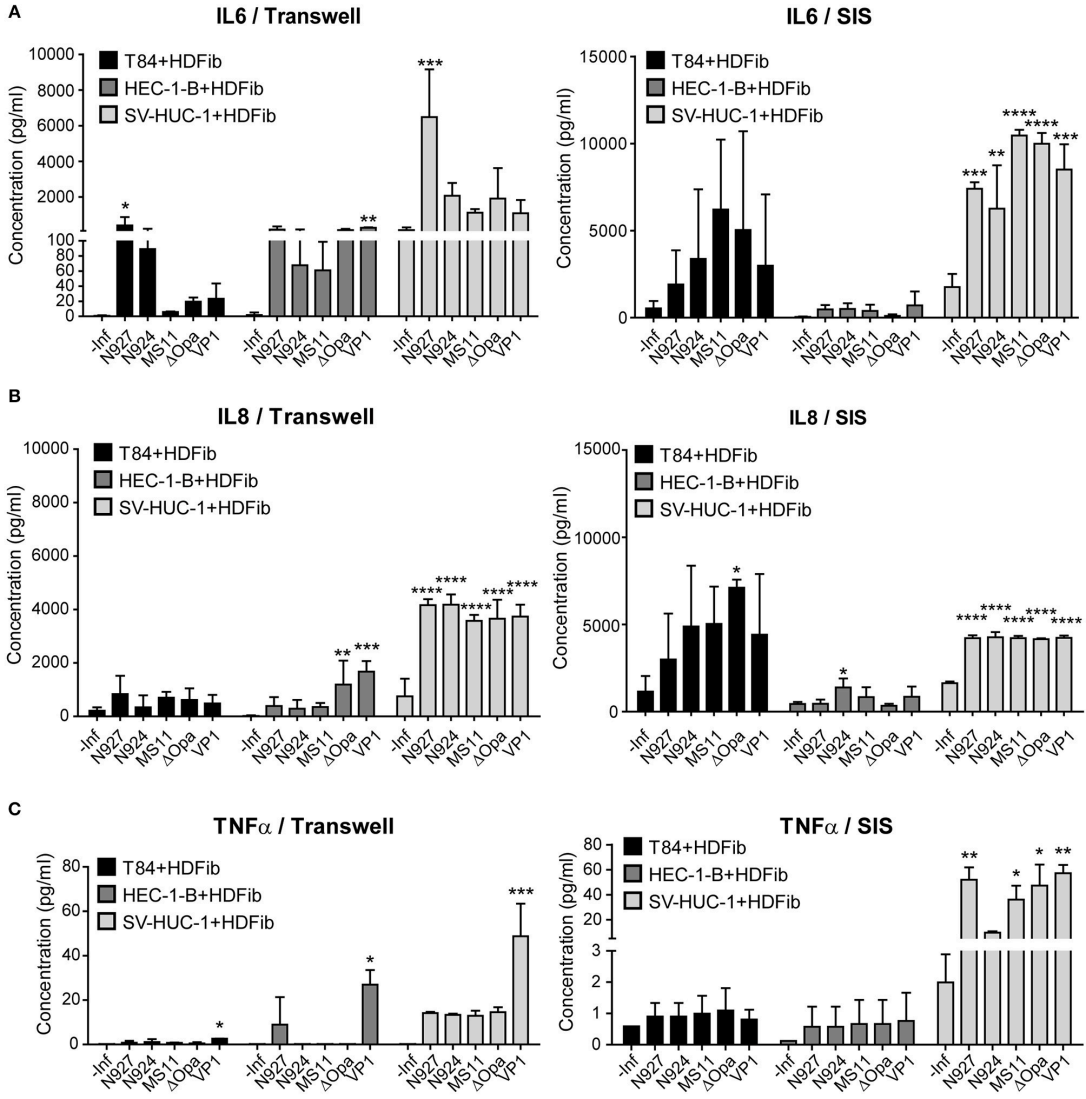
Measurement of the interleukin 6 and 8 and TNFα production of the Transwell® and SIS tissue models upon infection with *N. gonorrhoeae*. **(A–C)** Tissue models were prepared on Transwell® inserts or SIS as described for the Figure 1 and after maturation infected with different strains of *N. gonorrhoeae* for 24 h. Medium was collected and the concentration measurement of IL6, IL8, and TNFα was performed using Luminex assay. The graphs represent mean values ± SD from at least three independent replicates. Statistical significance of the changes in interleukin concentration in comparison to non-infected control was tested by one-way ANOVA: *p ≤ 0.05, **p ≤ 0.01, ***p ≤ 0.001, ****p ≤ 0.0001.

To determine the tissue response to infection with *N. gonorrhoeae*, we have measured the amount of pro-inflamatory cytokines IL-6 and TNFα, and chemokine IL-8 released into the apical medium 24 h post infection. We assessed the levels in both Transwell® and SIS scaffold models. Upon infection, SIS models produced much more IL-6 in comparison to Transwell® models. Tissue models with T84 and HEC-1-B cells produced less IL-6 than those with SV-HUC-1 cells, and we observed no greater differences between the bacterial strains and derivatives used for infection, except for N927 in the Transwell® model (Figure 8A).

The production of IL-8 was somewhat greater for SIS scaffold models in comparison to Transwell® models, especially for T84 cells. Again, we observed that uroepithelial models using SV-HUC-1 cells produced more IL-8 than those using other cells, but also that their response was highly significant. Here as well we observed no differences for different gonococcal strains and derivatives (Figure 8B).

We detected relatively low levels of TNFα expression in infected tissue models, which were higher for SIS scaffold than for Transwell® models. SV-HUC-1 models produced more TNFα upon infection, and N927 and VP1 strains were the most potent inducers of TNFα secretion (Figure 8C). Overall, our results show that SIS models are capable of secreting more IL-6, IL-8, and TNFα when challenged by *N. gonorrhoeae* than Transwell® models, that SV-HUC-1 uroepithelial cells react more pronounced to infection than T84 or HEC-1-B cells, and that there are no major differences in cytokine levels produced in response to different gonococcal strains and derivatives.

## Discussion

Our goal in this work was to mimic human mucosal and uroepithelial tissues, which represent the site of initial contact with *N. gonorrhoeae* and other sexually transmitted pathogens. Using these models, we studied different aspects of infection including bacterial attachment and transmigration to deeper tissue layers, destruction of the epithelial barrier, and elicited inflammatory response.

One of the main characteristics of successful epithelial tissue models is polarization of cells and formation of tight junctions, a process that go hand in hand. Polarization of cells does not only imply different protein content in the apical vs. the basolateral area of the plasma membrane, but includes alteration in lipid composition, most notably of phosphoinositides (Wang and Margolis, 2007). All this contributes to significant differences in the interaction of bacteria with polarized cells in comparison to classical 2-D tissue culture. So far, polarized epithelial cell models for research of gonococcal infection have been generated using Transwell® membranes or similar artificial porous supports, as exemplified by T84 and HEC-1-B Transwell® models (Stein et al., 2015; Wang et al., 2017). We aimed at increased model complexity by not only inducing the polarization of cells, but also by reconstructing the underlying layer of connective tissue. To this purpose we employed SIS scaffold, which represents an acellular biological extracellular matrix derived from porcine small intestinal submucosa. Primary constituents of SIS are collagen, elastin and fibronectin, but different growth factors are present, as well. The pore size of the SIS scaffold ranges from 20 to 30 μm, in comparison to only 3 μm in Transwell® membranes (Shi and Ronfard, 2013). We introduced fibroblasts to SIS scaffold to mimic the connective tissue. The presence of fibroblasts in addition to epithelial cells has been shown to have a positive effect on cell polarization due to the secretion of different components, which are important for the basal membrane formation (Steinke et al., 2014).

We measured the barrier integrity by TEER and FITC-dextran permeability assays, obtaining somewhat different results for TEER than already reported (Stein et al., 2015), and yet in concordance with results published by other groups (Navabi et al., 2013). TEER values, however, are reported to widely differ depending on the temperature, medium and type of electrode used for measurement (Srinivasan et al., 2015). Considering that TEER values we obtained were consistent and were related to low permeability of tissue models to FITC-dextran (Figure 1), as well as positive morphological characteristics (Figures 2–4), we presumed that tissue models were mature when they reached ~320 Ω^*^cm^2^ for T84 cells, ~160 Ω^*^cm^2^ for HEC-1-B, and ~135 Ω^*^cm^2^ for SV-HUC-1 cells in Transwell® models and ~340 Ω^*^cm^2^ for T84, ~180 Ω^*^cm^2^ for HEC-1-B and ~135 Ω^*^cm^2^ for SV-HUC-1 cells in SIS scaffold models. Our results therefore showed generally higher TEER values and two to three times lower FITC-dextran permeability for mature SIS models in comparison to Transwell® models in spite of the larger pore size (Figure 1). This is probably to be contributed to the presence of the basement membrane and fibroblasts in a thick layer of collagen and elastin fibers that constitute SIS as opposed to relatively thin polycarbonate membrane in Transwell® inserts.

Morphological characterization of SIS models shows that the cells polarize, as demonstrated by the presence of tight junctions and microvilli on the surface (Figures 3, 4). The mucus production is also present as observed in the form of a relatively thin mucus layer, which, in addition to mucus bubbles, has already been reported to be characteristic for polarized T84 cells (Navabi et al., 2013). Both HEC-1-B and SV-HUC-1 cells also exhibit a thin Muc1 layer (Figure 2B). Such layer for HEC-1-B models is comparable to the one identified in the mouse uterine tissue (DeSouza et al., 1999). The mucin layer in general and Muc1 in particular play a key role in protection of female reproductive tract from microbial infection (DeSouza et al., 1999). Therefore, it would be of interest to observe if and how the expression of mucin genes alters in tissue models in response to infection, as was done for human endocervical epithelial 3D tissue models (Radtke et al., 2012).

We used SIS models to study the outcome of infection depending on different pathogenicity factors in *N. gonorrhoeae* and different cell types. We could reproduce previously made observations that the lack of Opa proteins in *N. gonorrhoeae* enhances the transmigration of bacteria across the layer of polarized epithelial cells (Stein et al., 2015). Indeed, in Transwell® models after 6 h of incubation ΔOpa were by far the fastest bacteria to transmigrate (Figure 5A). This observation, however, depended on the model used—in the SIS scaffold model, the clinical isolate VP1 (PorB_IA_, Opa^−^, Pili^−^) was faster than ΔOpa when it came to crossing of the tissue barrier (Figures 5B,C). The observed loss of Opa protein expression in VP1 upon exposure to SIS scaffold models might explain its transmigration efficiency (Supplemental Figure 4B). However, it is difficult to reconcile the relatively high transmigration of N927 and MS11 bacteria and their apparent switch to an Opa^+^ phenotype (Figure 5, Supplemental Figure 4B). This indicates that we are still far from understanding the connection between the Opa phenotype and the ability of gonococci to transmigrate through tissue layers.

Pili play a central role in the attachment of gonococci to the tissue during infection (Punsalang and Sawyer, 1973). Although we selected MS11 and ΔOpa bacteria for the presence of pili, our later western blots showed that none of the bacteria expressed pilin either at the point of infection or afterwards (data not shown). Therefore, we are not at this stage able to discuss the observed differences in adherence and transmigration as a consequence of the piliation status. Also, we infected the tissue models under static conditions, and the importance of pilus might become obvious mostly when the shear stress is introduced through the circulation of the medium, which would require experimental adjustments and the usage of perfusion bioreactors, something that we plan to do in the future.

Overall, SIS models showed a much greater resilience to bacterial transmigration, giving us the opportunity to study the infection over the course of several days as opposed to several hours, as is the case for Transwell® models. For some strains the first bacteria transmigrating over the SIS scaffold models were detected only 6 days after infection, which can probably be contributed to the presence of the connective tissue-like layer beneath the epithelial cells. We could also show that the epithelial cell type influenced bacterial transmigration, with SV-HUC-1 cells allowing the lowest numbers of bacteria to traverse the barrier. This could be related to the flat appearance of these cells that overlay each other and do not offer bacteria an easy access to the area of cell-cell contacts (Figure 4).

The observed differences among the strains and tissues might be at this point discussed in the light of the question whether bacteria invade the cells and cross the tissue barrier through transcytosis, or whether they transmigrate between the cells and require destruction of the cell junctions for crossing of the tissue barrier. Our microscopy data do not indicate uptake of the gonococci by the epithelial cells, but rather support the localization of the bacteria in the area of cell-cell contacts (Figure 7, Supplemental Figure 3). Treatment of the SIS T84 tissue models with gentamicin from the apical and basolateral side 6 and 144 h after infection showed that all bacteria were efficiently killed (data not shown). This would not be the case if the bacteria were protected by being inside the cells. Therefore, it seems more likely that bacteria mostly cross the tissue models through transmigration, although more detailed experiments would be necessary to answer this question with certainty.

Interestingly, although *N. gonorrhoeae* are sensitive when cultured on plates or in liquid medium and are prone to autolysis (Garcia and Dillard, 2006), when in contact with SIS models, viable bacteria could be collected throughout the whole 6 day period of infection experiments (Figures 5, 7). Our results show that whereas VP1 was able to successfully colonize the tissue and increase its numbers rapidly throughout 6 days of infection, N924 were incapable of doing that, in spite of the comparable growth in liquid culture (Figure 7, Supplemental Figure 4). In this aspect as well, SIS models offer valuable tools for understanding gonococcal interaction with host tissues.

When comparing the permeability and induced cell death of infected models, whereas there is a relatively uniform change in the permeability of Transwell® models independently of the bacterial strain or derivative used (Figure 6A), SIS models enable us to observe fine differences in the permeability changes depending on pathogenicity factors that bacteria exhibit (Figure 6B). Interestingly, the number of transmigrated bacteria does not entirely correlate to the changes in barrier permeability, which is seen on the example of N927 gonococci in SIS scaffold HEC-1-B and SV-HUC-1 models (Figure 6B). It would appear that they are capable of increasing the barrier permeability through destruction of cell-cell junctions or by host cell death, but remaining at the same time associated with the tissue.

Confocal fluorescence microscopy offers a good tool for observing the integrity of the SIS scaffold models and the interaction of epithelial cells with bacteria. In our experiments we detect bacteria only at the surface of the models. It is however important to note that during the staining process there is a gradient of dyes and antibodies throughout the tissue model, which means that the staining of the structures deeper below the surface is increasingly weaker. To address this problem, staining should be improved and other microscopy techniques could be implemented that would enable us to visualize the tissue throughout its entire thickness.

The results of the Luminex assay showed that *N. gonorrhoeae* significantly induced the production of interleukins in SIS tissue models. The basal levels of produced cytokines were low in tissue models without infection and they increased after exposure to the gonococci. Moreover, the levels of inflammatory mediators were cell and bacteria type dependent. Here, we also see clear differences in comparison to the Transwell® models, in terms of different pattern of cytokine response to different gonococcal strains and derivatives and in quantities of cytokines produced. The latter might be the consequence of the presence of fibroblasts in the SIS tissue models. Although we have also added fibroblasts to the basal side of the Transwell® membrane in attempt to mimic similar cell content as in SIS models, it is possible that the SIS scaffold environment better supports establishment and multiplication of fibroblasts than the Transwell® membrane. Our results also imply much stronger and reproducible response of male urothelium to infection with gonococci than it is the case with endometrial epithelium represented by the HEC-1-B cells, which might be the explanation for differences in the course of infection in males and females (Edwards and Apicella, 2004).

Several publications show that the shedding of epithelial cells takes place during gonococcal infection. *N. gonorrhoeae* is reported to cause exfoliation of columnar epithelial cells of the human endocervix in the model of tissue explants, and of polarized T84 cells in the Transwell® model (Wang et al., 2017). Shedding of urethral epithelial cells has also been observed in the samples obtained from male gonorrhea patients (Apicella et al., 1996). In this work, we were not able to reliably quantify detachment of the cells from the surface of the SIS scaffold models, but we did observe tissue destruction and the disturbance of the tight junction (Figures 6, 7). This effect, however, depended on the bacterial strain, because the MS11 derivative N924, lacking three major virulence factors (pilus, Opa and PorB_IA_) had a significantly milder effect on the tissue integrity than the clinical isolate VP1.

For further improvement of the SIS scaffold models there are couple of important aspects to consider. One is the introduction of primary cells and the other is the hormone responsiveness of the modeled tissues. The availability of primary epithelial cells from the urogenital tract is restricted and their culturing is of limited duration, which might be overcome by the usage of organoid technology (Kessler et al., 2015; Boretto et al., 2017) or stem cells (Wu et al., 2011). The role of the tissue-specific stromal cells is also of significance, especially for endometrial tissue, where stromal cells contribute to the growth of epithelial cells as well as to the tissue response to hormones (Arnold et al., 2001; Bläuer et al., 2005). Such improvement of the models would enable us additionally to address and study the relationship between the hormonal status of the host and the infection.

In conclusion, we established three independent 3D co-cultured tissue models of human HEC-1-B, SV-HUC-1, and T84 with human fibroblast cells on a biological decellularized scaffold. To our knowledge, this is the first report on establishing a 3D tissue model including co-culturing of epithelial and fibroblast cells to study neisserial infection. Our models provide physiologically relevant conditions containing both the connective tissue with fibroblasts and polarized epithelial monolayer of mucosal surfaces, and as such represent a significant advance in modeling of *N. gonorrhoeae* infection.

## Materials and Methods

### Cell Lines

HEC-1-B, the human endometrial adenocarcinoma cell line (ATCC® HTB113™), and human dermal fibroblasts (HDFib), isolated according to the published protocol from foreskin biopsies of healthy donors (Pudlas et al., 2011), were cultured in Dulbecco's Modified Eagle Medium (DMEM) (Gibco/Thermo Fisher scientific, Massachusetts, USA). SV-HUC-1 (ATCC® CRL-9520™), the human ureter uroepithelial SV40 immortalized cells, were cultured in Ham's F12 Nutrient Mixture (Gibco/Thermo Fisher scientific, Massachusetts, USA) and T84, the human colorectal carcinoma cells (ATCC® CCL-248™) were cultured in DMEM/F12 (Gibco/Thermo Fisher scientific, Massachusetts, USA). All media were supplemented with 10% heat-inactivated fetal calf serum (FCS) (Sigma/Merck, Darmstadt, Germany) and 1% Penicillin/Streptomycin (Gibco/Thermo Fisher scientific, Massachusetts, USA).

### Generation of the Human 3D Tissue Cell Models

SIS scaffold models: Preparation of porcine small intestinal submucosa scaffold and decellularization were done according to the established protocol (Schweinlin et al., 2016). Pieces of SIS scaffold were mounted on the plastic 6.5 mm diameter cell crowns and 100,000 fibroblasts were seeded on the apical side of each cell crown in the appropriate medium. After 48 h, 300,000 epithelial cells were seeded on the apical side of the model. Tissue models were cultured under submerged static conditions or with shaking (only in experiment shown in Figure 1) on an orbital shaker at 25 rpm for 12 days in the case of T84 or SV-HUC-1 cells, or 14 days in the case of HEC-1-B cells at 37°C/5% CO_2_ in the tissue culture incubator. The medium was exchanged every 2 days. In case where different medium was required for fibroblasts and epithelial cells, the models were cultured in the medium consisting of 50:50 fibroblast:epithelial cell medium. After maturation, tissue models were either fixed for further staining or the medium was exchanged for the one lacking antibiotic to enable the infection 24 h later.

Transwell® models: 6.5 mm diameter, 3 μm pore size polyester Transwell® inserts (Corning, Lowell, MA, USA) were coated with rat tail collagen type I, 100,000 fibroblasts were seeded on the basal side and 48 h later 200,000 epithelial cells were seeded on the apical side of the Transwell® membrane. Models were grown for 10 days under submerged static conditions at 37°C/5% CO_2_ in the tissue culture incubator prior to further handling (fixation and staining or infection 24 h after medium change).

### Barrier Integrity

We used TEER as a measurement for the barrier integrity of the epithelial cell monolayer (Srinivasan et al., 2015). We considered TEER values bellow the one for the empty SIS scaffold (between 80 and 90 Ω^*^cm^2^) or empty Transwell® insert (between 50 and 60 Ω^*^cm^2^) as a background. TEER was measured using Millicell® ERS-2 Volt-Ohm Meter. In addition, the integrity of the monolayer was assessed using 4 kDa FITC-dextran (Sigma, Darmstadt, Germany) permeability assay after 14 days of cultivation for HEC-1-B and 12 days of cultivation for T84 and SV-HUC-1 cell lines. To this purpose, 0.25 mg/ml FITC-dextran was dissolved in cell culture medium and filtered. The medium was removed from the apical and basal sides of the cell crown or Transwell®. One milliliter of fresh medium was added to the basal side, and 300 μl of FITC-dextran-containing medium to the apical side. After 30 min of incubation, 200 μl from the lower compartment were collected into a 96 well plate and fluorescence was analyzed using TECAN reader (absorption 490 nm, emission 525 nm). The results were normalized to the sample with an empty SIS scaffold or Transwell® membrane.

### *Neisseria gonorrhoeae* Strains and Culture Conditions

*Neisseria gonorrhoeae* N927 (PorB_IA_, Opa^−^, Pili^−^), N924 (PorB_IB_, Opa^−^, Pili^−^), MS11 (PorB_IB_, Opa^+^, Pili^+^), MS11 ΔOpa (PorB_IB_, Opa^−^, Pili^+^), VP1 clinical strain (PorB_IA_, Opa^−^, Pili^−^), N931 (PorB_IB_, Opa^50^, Pili^−^), and N313 (PorB_IB_, Opa^57^, Pili^−^) were grown on GC agar plates (Thermo Fisher Scientific) supplemented with 1% vitamin mix for 14–17 h at 37°C in 5% CO_2_. For growth curve measurements, bacteria were grown overnight on GC-agar plate, resuspended in PPM medium to OD_550_ = 0.2 and allowed to grow to OD_550_ between 0.5 and 0.6. All cultures were diluted to OD_550_ = 0.1 in PPM medium (15 g Proteose peptone; 5 g sodium chloride; 0.5 g soluble starch; 1 g potassium dihydrogen phosphate; 4 g dipotassium hydrogen phosphate for 1 l; pH 7.2; 1% vitamin mix, 0.5% sodium hydrogen carbonate, 10 mM magnesium chloride, sterilized by filtration) and incubated with shaking at 37°C. OD_550_ was measured at different time points to assess growth.

### Histology

Tissue models were fixed in 4% paraformaldehyde. After paraffin embedding, samples were sectioned to 6 μm thickness. Hematoxylin and eosin staining was performed after the deparaffinization process in xylene (Steinke et al., 2014; Schweinlin et al., 2017).

### Immunofluorescence Analysis

Four percentage paraformaldehyde were used to fix the tissue models for 2 h on cell crowns. The tissue models were then washed with phosphate buffered saline (PBS), permeated using 1% Saponin (Sigma, Darmstadt, Germany), blocked with 1% BSA in PBS and decorated with primary antibodies overnight. This was followed by decoration with fluorophore-coupled secondary antibodies (Dianova, Hamburg, Germany), Phalloidin (MoBiTec, Göttingen, Germany), DAPI (Sigma, Darmstadt, Germany), and mounting using Dako (Agilent, Santa Clara, United States). Z-stacks of images were obtained through 25 μm from the top of the monolayer using Leica SP5 and processed by FIJI (Schindelin et al., 2012) and FIJI Plugin 3D Viewer (Schmid et al., 2010).

### Scanning Electron Microscopy/Transmission Electron Microscopy

Tissues were fixed for 1 h with 2.5% glutaraldehyde (50 mM cacodylate [pH 7.2], 50 mM KCl, and 2.5 mM MgCl_2_) at room temperature for TEM and 6.5% glutaraldehyde for SEM microscopy. Further preparations and analysis of the samples proceeded as already described (Spiliotis et al., 2008; Ott et al., 2012) using JEM-2100 and JSM-7500F JEOL microscopes.

### LDH Assay

Cytotoxicity Detection Kit (Roche) was used in order to quantify the cell death and cell lysis rate based on lactate dehydrogenase (LDH) activity in the supernatants. The experiment was performed according to the manufacturer's instructions.

### Bacterial Infection and Colonization Assays

After evaluating the models for barrier integrity, the infection was performed in the phosphate-free HEPES medium, as described before (Kühlewein et al., 2006), using MOI 20. To assess the transmigration of *N. gonorrhoeae* across the polarized epithelial monolayer after different infection time points, the medium from the bottom compartment was collected. Fifty microliter of medium were plated directly onto the GC agar plate in case of Transwell® inserts, or the medium was centrifuged shortly at 100 × g, the pellet was resuspended in remaining 50 μl of medium and plated on GC agar.

For assessment of bacterial adhesion, the infected tissues were incubated for 30 min with 1% Saponin (Sigma, Darmstadt, Germany) and dilution series were cultured on GC agar.

### Cytokine Quantification

Highly sensitive customized Luminex assay kit was used in order to detect and quantify TNFα, IL-6, and IL-8 in the medium from the apical compartment of Transwell® or SIS tissue models.

### Antibodies

Antibodies used in the work are anti-ZO1 and anti-E-Cadherin (Proteintech, Manchester, United Kingdom), anti-Fibroblast (Novusbio, Colorado, United states), anti-Muc1 (Santa Cruz, Texas, United states), and anti-*N. gonorrhoeae* (USBiological, Swampscott, Massachusetts, USA). *N. gonorrhoeae* Omp85 antibodies were raised in rabbits against the full-length His-tagged protein. The pan-Opa antibody was a kind gift from Christof Hauck and has been already described (Achtman et al., 1988).

### Statistical Analyses

Statistical analyses were performed with one-way ANOVA, Tukey's multiple comparison test, using GraphPad Prism Software (GraphPad Software, Inc.).

## Data Availability

All datasets generated for this study are included in the manuscript and/or the Supplementary Files.

## Author Contributions

VK-P, TR, HW, MSc, and MSt designed the experiments. MH and TY conducted the experiments. MH, TR, and VK-P analyzed the results. HW, MSc, and MSt provided the material. MH and VK-P wrote the manuscript.

### Conflict of Interest Statement

The authors declare that the research was conducted in the absence of any commercial or financial relationships that could be construed as a potential conflict of interest.

## Supplementary Material

Supplemental Figure 1 Cells used for generation of tissue models. Cells (HDFib, primary human dermal fibroblasts; T84, human colon carcinoma cell line; HEC-1-B, human endometrial adenocarcinoma cell line; SV-HUC-1, human uroepithelium SV40 immortalized cell line) were grown in tissue culture flasks and the images were made using inverted phase contrast microscope. Scale bar is 100 μm.

Supplemental Figure 2 ZO1 staining of SV-HUC-1 SIS scaffold mucosal tissue model. Epithelial/fibroblast co-culture tissue models were prepared corresponding to Figure 3. After fixing and decorating with ZO1 antibody (red channel) and DAPI (blue channel), the samples were analyzed using fluorescence confocal microscopy. Shown are Z projections of several Z-stack images. Scale bar is 25 μm.

Supplemental Figure 3 Scanning electron microscopy of HEC-1-B SIS mucosal tissue models. **(A)** Tissue models on SIS scaffold were generated as described in Figure 1 and analyzed by scanning electron microscopy. **(B)** The models as in **(A)** were infected for 24 h with *N. gonorrhoeae* strain N927 and analyzed by scanning electron microscopy.

Supplemental Figure 4 **(A)** Growth curve of *N. gonorrhoeae* strains and derivatives used to infect tissue models. Bacteria were grown overnight on GC-agar plate, resuspended in PPM medium to OD_550_ = 0.2 and grown to OD_550_ = 0.5 to 0.6. All cultures were diluted to OD_550_ = 0.1 in PPM medium and allowed to grow, with OD_550_ being measured at indicated time points. The graph represents mean values ± SD from three independent replicates. **(B)** Control strains (N931 expressing Opa50 and N313 expressing Opa57), as well as bacteria collected from the basolateral side after 6 days of infection of the indicated SIS scaffold tissue models were centrifuged, lysed in Lämmli buffer and analyzed by SDS-PAGE and western blot, using pan-Opa and Omp85 antibodies.

Supplemental Figure 5 Traversing of the empty and SIS-HDFib scaffold by different *N. gonorrhoeae* strains and derivatives. Empty SIS scaffold was mounted on cell crowns in cell culture medium. 100,000 HDFib were introduced to the scaffold 2 days prior to infection. Infection was performed in the HEPES medium at MOI 20 and was allowed to proceed for 7 h. 25 μl samples were collected from the basolateral compartment at indicated time points and plated with serial dilutions on GC agar plates for CFU counting. CFUs were counted up to the maximum of 100,000. The graphs show mean values ± SD from two independent replicates.

Supplemental Figure 6 Changes in the permeability of the SIS scaffold mucosal tissue models after infection with *N. gonorrhoeae*. SIS scaffold tissue models were generated and infected as described for the Figure 5. The barrier permeability was measured using 4 kDa FITC-Dextran assay at indicated time points. The graphs show mean values ± SD from at least three independent replicates.

Supplemental Movies 1–3 3D reconstructed images after confocal microscopy of SIS mucosal tissue models. SIS models were generated as described for Figure 3 using T84 cells (Supplemental Movie 1), HEC-1-B cells (Supplemental Movie 2), or SV-HUC-1 cells (Supplemental Movie 3), fixed on cell crowns, then decorated using anti-zonula occludens 1 (ZO1) antibody, phalloidin (actin), and DAPI. Z-stacks were generated by fluorescence confocal microscope from the top of the epithelial layer to the beginning of the collagen scaffold and reconstructed using FIJI.

Supplemental Movies 4–7 3D reconstructed images after confocal microscopy of infected T84 SIS tissue models. Tissue models with T84 epithelial cells, as described in Figures 5, 7, were prepared and infected with *N. gonorrhoeae* strains N924 for 24 h (Supplemental Movie 4), N924 for 144 h (Supplemental Movie 5), VP1 for 24 h (Supplemental Movie 6), or VP1 for 144 h (Supplemental Movie 7). The infected models were fixed on cell crowns and decorated using anti-zonula occludens 1 (ZO1), anti-*N. gonorrhoeae* antibody, and DAPI. Z-stack images were made using fluorescence confocal microscope beginning at the top of the epithelial layer to the beginning of collagen scaffold and reconstructed using FIJI.
