# Supplementary figures and images for: Biomimetic Human Tissue Model for Long-Term Study of Neisseria gonorrhoeae Infection
Source: Front Microbiol. 2019 Jul 31;10:1740. doi: 10.3389/fmicb.2019.01740 (PMC6685398; doi:10.3389/fmicb.2019.01740)

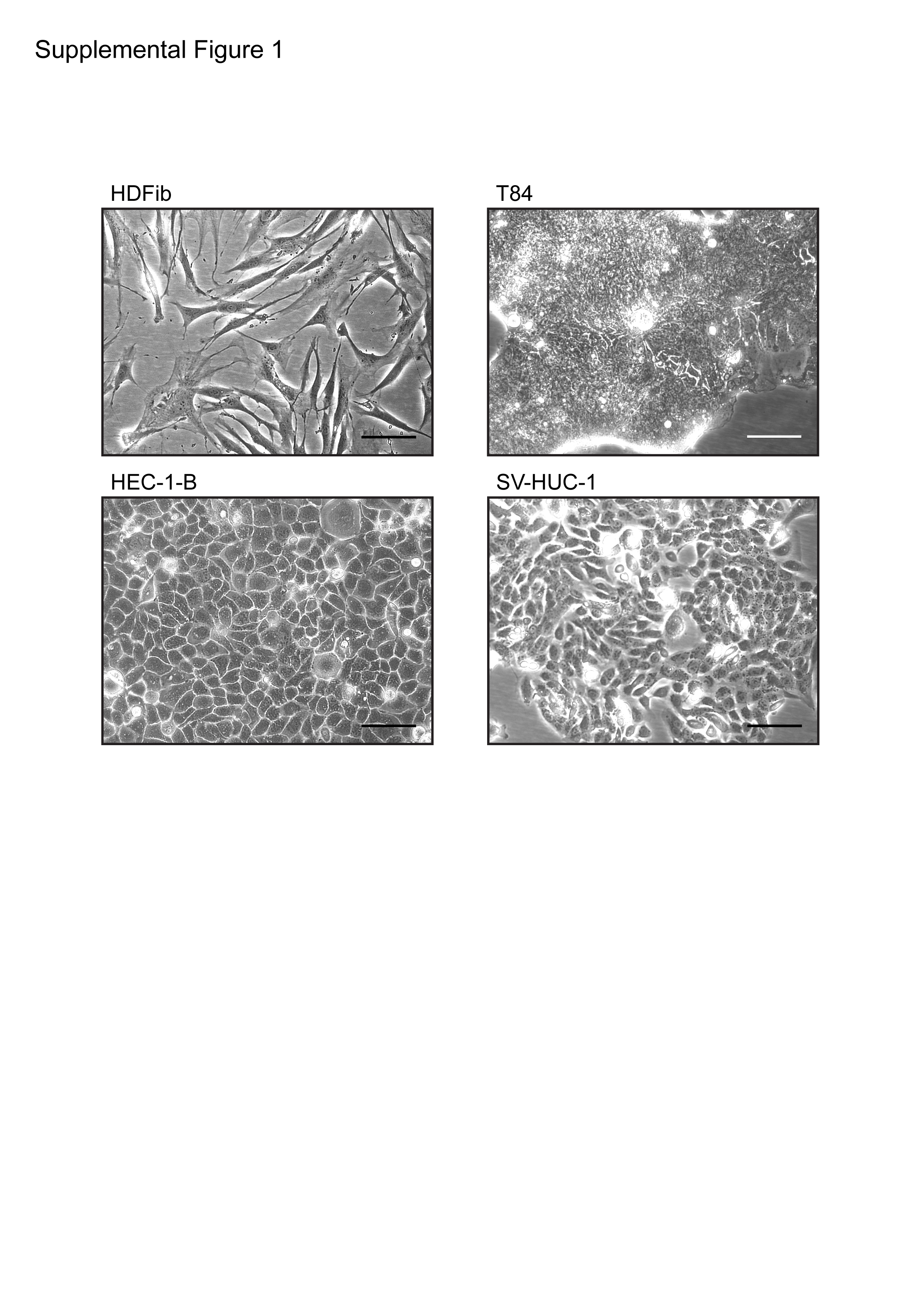

Supplement: Supplemental Figure 1 — Cells used for generation of tissue models. Cells (HDFib, primary human dermal fibroblasts; T84, human colon carcinoma cell line; HEC-1-B, human endometrial adenocarcinoma cell line; SV-HUC-1, human uroepithelium SV40 immortalized cell line) were grown in tissue culture flasks and the images were made using inverted phase contrast microscope. Scale bar is 100 μm. [file Image_1.TIF]

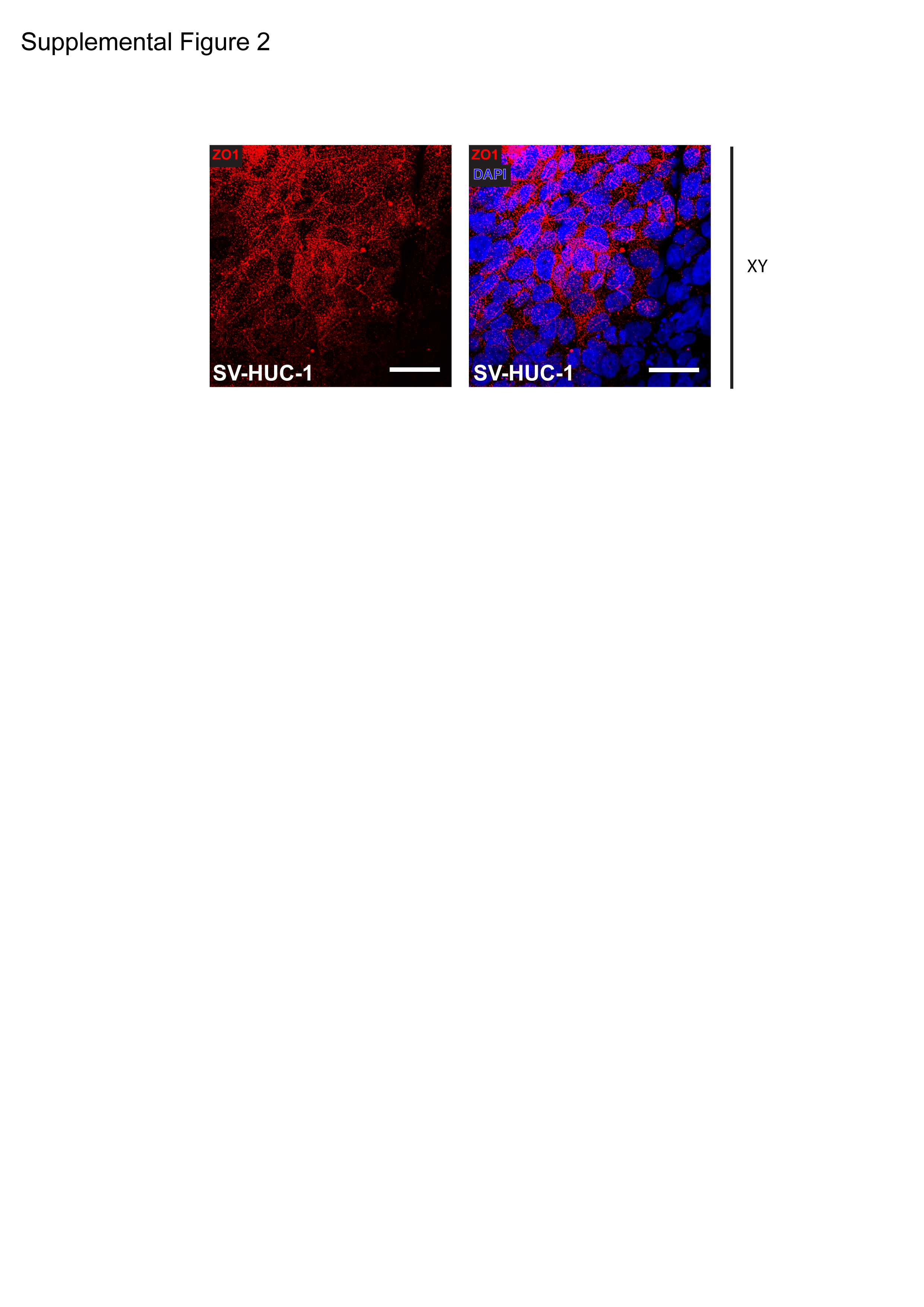

Supplement: Supplemental Figure 2 — ZO1 staining of SV-HUC-1 SIS scaffold mucosal tissue model. Epithelial/fibroblast co-culture tissue models were prepared corresponding to Figure 3. After fixing and decorating with ZO1 antibody (red channel) and DAPI (blue channel), the samples were analyzed using fluorescence confocal microscopy. Shown are Z projections of several Z-stack images. Scale bar is 25 μm. [file Image_2.TIF]

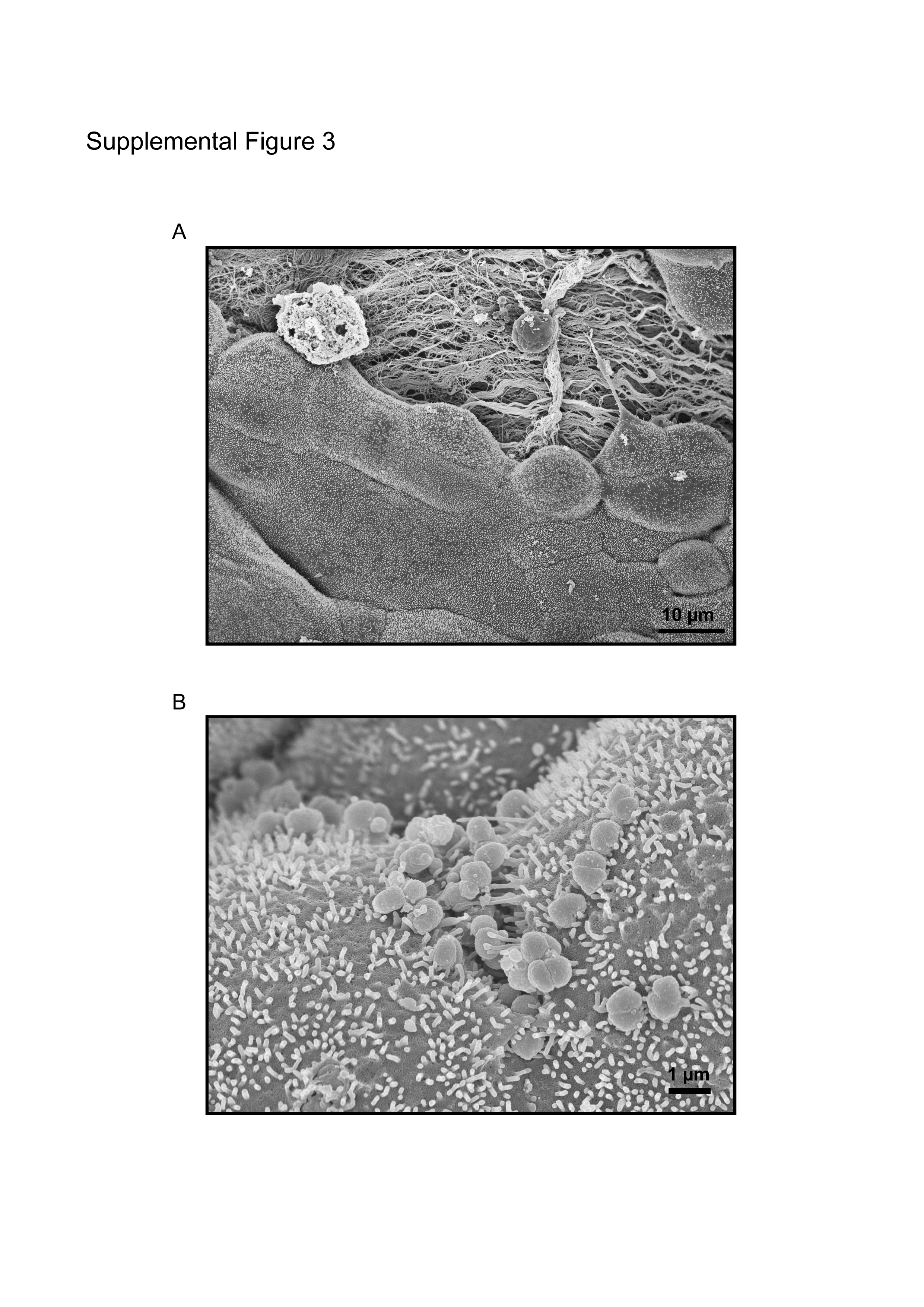

Supplement: Supplemental Figure 3 — Scanning electron microscopy of HEC-1-B SIS mucosal tissue models. (A) Tissue models on SIS scaffold were generated as described in Figure 1 and analyzed by scanning electron microscopy. (B) The models as in (A) were infected for 24 h with N. gonorrhoeae strain N927 and analyzed by scanning electron microscopy. [file Image_3.TIF]

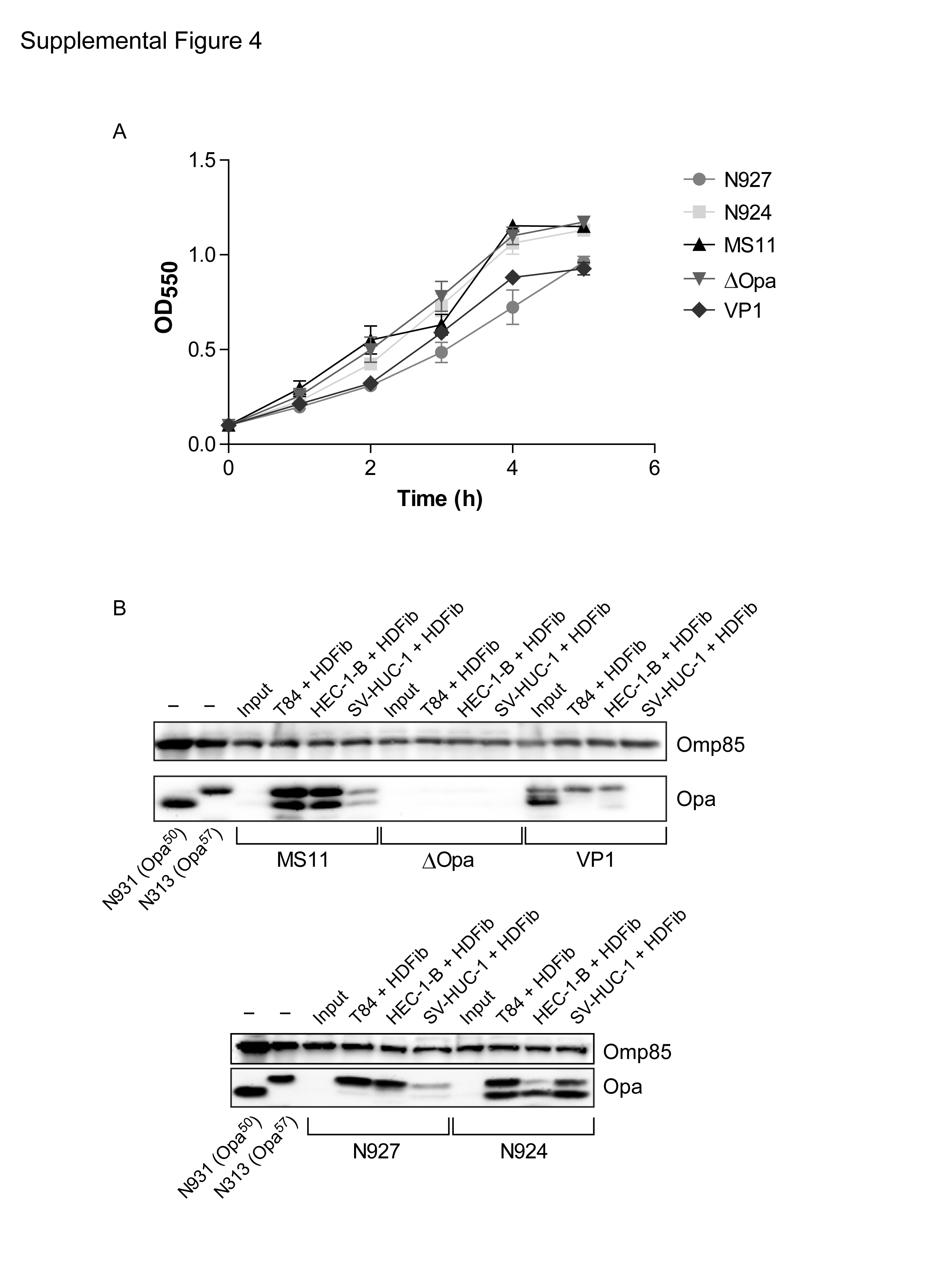

Supplement: Supplemental Figure 4 — (A) Growth curve of N. gonorrhoeae strains and derivatives used to infect tissue models. Bacteria were grown overnight on GC-agar plate, resuspended in PPM medium to OD550 = 0.2 and grown to OD550 = 0.5 to 0.6. All cultures were diluted to OD550 = 0.1 in PPM medium and allowed to grow, with OD550 being measured at indicated time points. The graph represents mean values ± SD from three independent replicates. (B) Control strains (N931 expressing Opa50 and N313 expressing Opa57), as well as bacteria collected from the basolateral side after 6 days of infection of the indicated SIS scaffold tissue models were centrifuged, lysed in Lämmli buffer and analyzed by SDS-PAGE and western blot, using pan-Opa and Omp85 antibodies. [file Image_4.TIF]

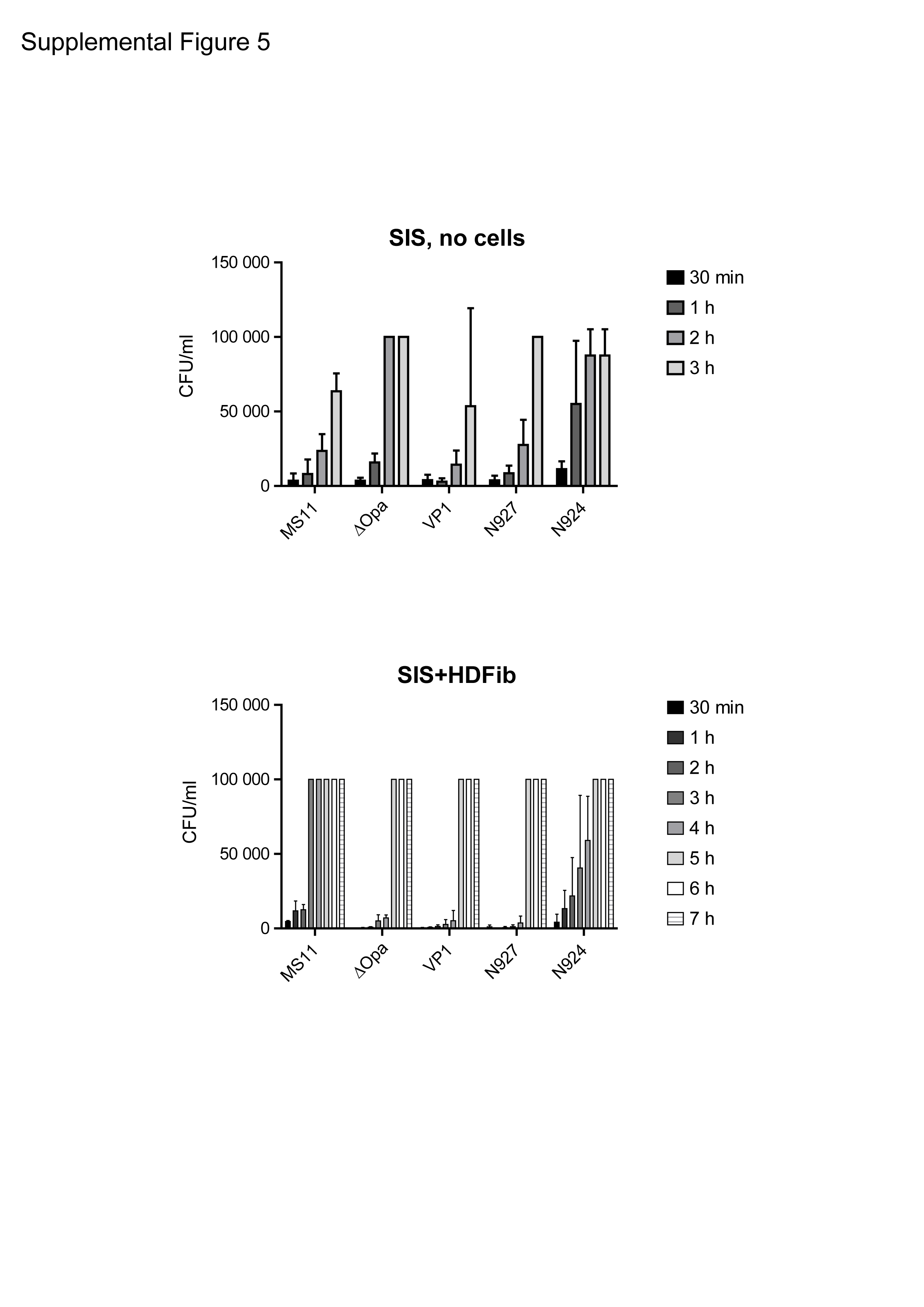

Supplement: Supplemental Figure 5 — Traversing of the empty and SIS-HDFib scaffold by different N. gonorrhoeae strains and derivatives. Empty SIS scaffold was mounted on cell crowns in cell culture medium. 100,000 HDFib were introduced to the scaffold 2 days prior to infection. Infection was performed in the HEPES medium at MOI 20 and was allowed to proceed for 7 h. 25 μl samples were collected from the basolateral compartment at indicated time points and plated with serial dilutions on GC agar plates for CFU counting. CFUs were counted up to the maximum of 100,000. The graphs show mean values ± SD from two independent replicates. [file Image_5.TIF]

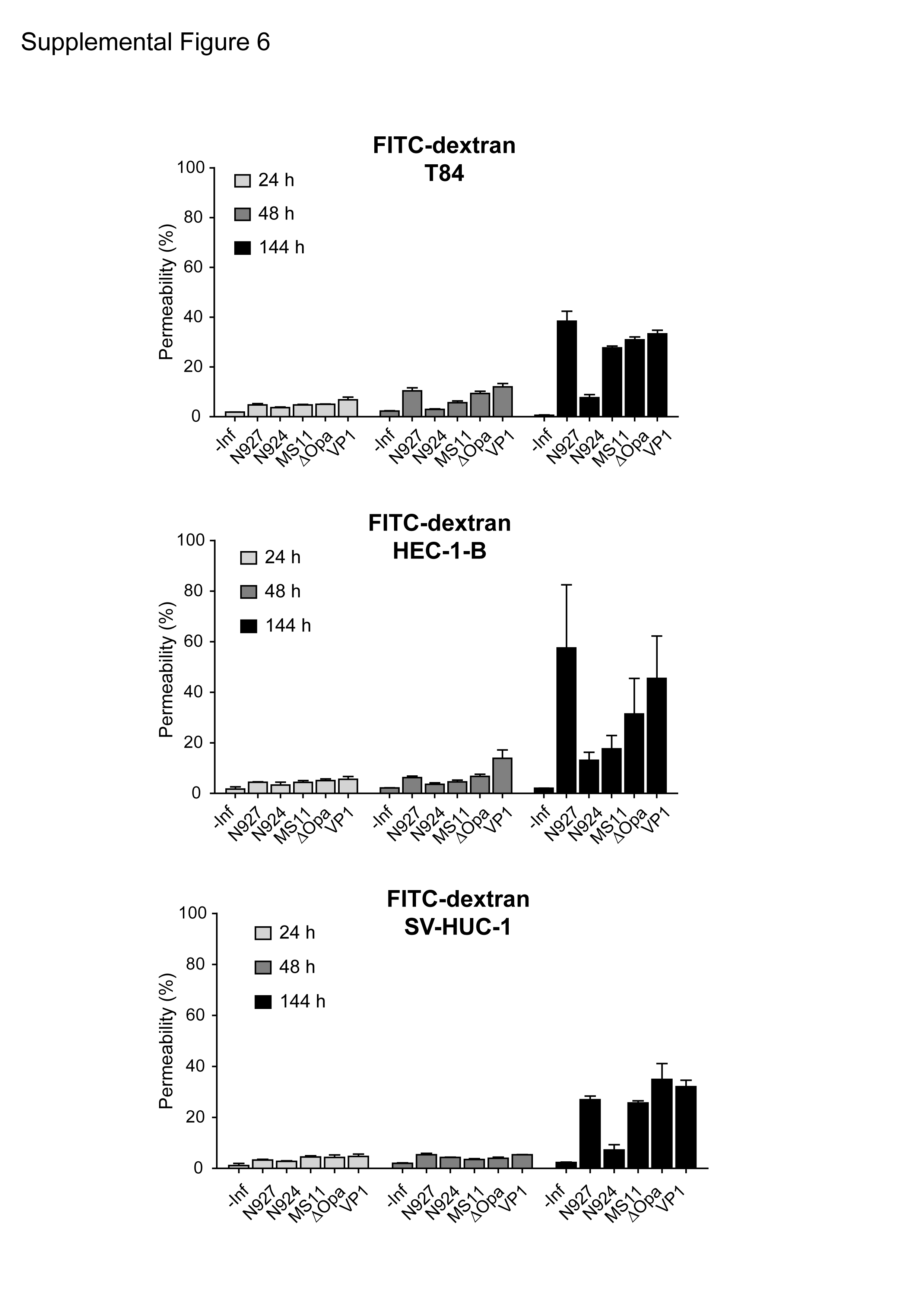

Supplement: Supplemental Figure 6 — Changes in the permeability of the SIS scaffold mucosal tissue models after infection with N. gonorrhoeae. SIS scaffold tissue models were generated and infected as described for the Figure 5. The barrier permeability was measured using 4 kDa FITC-Dextran assay at indicated time points. The graphs show mean values ± SD from at least three independent replicates. [file Image_6.TIF]
